# Plastic responses of *Plasmodium relictum* to competent and non-competent mosquito bites

**DOI:** 10.1098/rsbl.2025.0464

**Published:** 2025-11-05

**Authors:** Valentin Chauvin, Arnaud Berthomieu, Claire Loiseau, Christian N. Tchana, Frédéric Angelier, Ana Rivero

**Affiliations:** ^1^ MiVEGEC (University of Montpellier, CNRS, IRD), Montpellier, France; ^2^ Centre d’Etudes Biologiques de Chizé, CNRS, La Rochelle Université, UMR7372, Villiers-en-Bois, France

**Keywords:** avian malaria, phenotypic plasticity, gametocytes, disease vectors

## Abstract

Environmental unpredictability challenges the transmission success of vector-borne parasites like *Plasmodium*, whose fitness depends on synchronizing the production of transmission forms, called gametocytes, with vector availability. Although mosquito bites are known to trigger *Plasmodium* responses, it remains unclear whether parasites respond specifically to competent vectors or adopt a generalist strategy. We experimentally infected birds with the avian malaria parasite *Plasmodium relictum* and exposed them to bites from either a competent (*Culex quinquefasciatus*) or a non-competent (*Anopheles gambiae*) vector species. Both exposures induced a rise in parasitaemia, but the response was significantly stronger to the non-competent species. Host inflammatory, humoral and stress markers remained similar across mosquito treatments, implying that unidentified physiological cues may underlie the parasite’s response. No species-specific modulation of gametocyte conversion rate or sex ratio was observed. These findings suggest that *P. relictum* does not discriminate between mosquito species, instead employing a generalist, possibly bet-hedging strategy in response to any mosquito bite. Our results highlight the importance of mosquito community composition in shaping parasite transmission dynamics. If non-vector mosquitoes enhance *Plasmodium* transmission investment, shifts in vector assemblages due to climate change or control measures may have unanticipated effects on disease ecology.

## 1. Introduction

Unpredictable environments challenge all living organisms, including parasites. This is particularly true for vector-borne parasites, whose fitness depends on aligning their transmission strategies with the fluctuating availability of competent vectors [1]. Such ecological uncertainty exerts strong selective pressure for mechanisms that allow dynamic and timely adjustment of transmission-related traits. One such mechanism is adaptive phenotypic plasticity, the capacity to modulate life-history traits in real time in response to environmental cues [2].

Among parasites, *Plasmodium*, the causative agent of malaria, provides a well-documented example of such plasticity [3]. Its transmission success depends not only on within-host replication but also on the timely production of gametocytes, the only stage infectious to mosquitoes. The switch from asexual replication to male and female gametocyte production represents a major life history trade-off, as it diverts resources from proliferation to transmission [4]. Theoretical frameworks such as terminal investment [5], reproductive restraint [6], fertility insurance [7] and local mate competition (LMC) [8] offer predictions on how these traits may vary in response to environmental cues.

One such cue may be the bite of the mosquito itself. It has long been hypothesized that mosquito bites signal transmission opportunities to *Plasmodium* parasites [9]. Empirical studies support this idea: seasonal increases in vector abundance are associated with heightened gametocytogenesis [10], repeated mosquito exposure can enhance parasitaemia and transmission [11] and short-term increases in mosquito infectivity can occur within hours of mosquito contact [12].

Previous work has largely operated under the implicit assumption that *Plasmodium* responds to cues associated with mosquito bites that reliably signal the presence of a competent vector. In reality, hosts are exposed to a diverse community of mosquito species, many of which are not capable of transmitting the parasite. Whether *Plasmodium* can distinguish among these different mosquito species, and selectively mount responses to those that represent genuine transmission opportunities, remains an open and largely unexplored question.

Two possible scenarios could explain how the parasite navigates this challenge. First, *Plasmodium* may detect and respond specifically to bites from competent vectors. Alternatively, it may respond indiscriminately to any mosquito bite, a strategy that may be adaptive where vector species have overlapping temporal dynamics. Discriminating between these scenarios requires identifying the signals that parasites might use to detect mosquito bites. Mosquito saliva is the primary candidate as it contains a cocktail of bioactive molecules capable of inducing host immune, hormonal and inflammatory responses [13–15]. Saliva composition differs between mosquito species [16–18] and may elicit different physiological reactions in the host, both in terms of the local immune response and systemic physiological changes [19–21].

Here, we test whether bites from different mosquito species modulate gametocyte conversion rate and sex ratio in *Plasmodium,* two highly plastic traits, which are central to transmission success. Conversion rate reflects the balance between within-host replication and transmission and may range from reproductive restraint, where asexual replication is prioritized to maintain and prolong infection, to terminal investment, where gametocyte production is maximized to enhance transmission and facilitate escape from the host [4]. Gametocyte sex ratio, in turn, is shaped by LMC theory, which predicts female-biased allocation under high relatedness and high gametocyte density. Conversely, male production increases when female gametocyte fertilization is at risk due to low relatedness or low gametocyte densities, a strategy known as fertility insurance [8,22,23].

To address this question, we use the avian malaria parasite *Plasmodium relictum*, a highly prevalent and generalist parasite of wild passerine birds, whose main natural vectors are mosquitoes within the *Culex pipiens* complex [24]. Other confirmed vectors to date include additional *Culex* species and *Culicoides* species (MalAvi Database [25]). No confirmed records of *Anopheles* vectors of *P. relictum* exist, and previous experimental work showed that development of this parasite in *Anopheles* species is incomplete or inconsistent [26]. Previous studies have reported significant increases in blood parasitaemia and infectivity to mosquitoes in birds exposed to *Cx. pipiens* bites, yet these effects occur without detectable changes in gametocytaemia [11,12]. However, the accurate quantification and sexing of gametocytes in avian malaria has long been hindered by the limitations of conventional microscopy [27]. As a result, our understanding of the transmission traits driving the epidemiology and evolution of this parasite remains incomplete, despite its high virulence in naive host populations, and its significant impact on avian biodiversity in newly colonized regions [28–30]. Here, we overcome these challenges by using newly developed molecular markers that allow a more precise measurements of male and female gametocytes [27].

Birds infected with *P. relictum* were exposed to one of three treatments: no mosquito bites (control), bites from *Culex quinquefasciatus* (competent vector), or bites from *Anopheles gambiae* (non-competent vector). *An. gambiae* is a major vector of human malaria in Africa, but its geographic range does not overlap with that of *P. relictum pSGS1*, nor is it capable of transmitting this avian parasite. Although it exhibits a strong preference for human hosts [31], it readily feeds on birds under laboratory conditions.

We addressed the following questions: (i) do *P. relictum* respond plastically to mosquito bites by changing the number or sex ratio of gametocytes? (ii) Do these responses differ between vector and non-vector bites? (iii) Are host physiological responses to mosquito bites potential mediators of parasite plasticity? To explore the latter, we quantified two immune parameters in birds exposed to vector and non-vector bites: C-reactive protein (CRP, a marker of inflammation [32]) and IgY (the avian equivalent of mammalian IgG, which plays a central role in adaptive immunity [33]). In addition, we measured circulating levels of corticosterone, a stress hormone recently shown to enhance parasitaemia in birds [34].

Our working hypothesis is that mosquito bites function as proximal environmental signals, triggering increased reproductive investment in the parasite. Specifically, we predict a rise in conversion rate and a shift in sex ratio towards greater female production, reflecting reduced need for fertility insurance at higher gametocyte densities. Furthermore, we hypothesize that the magnitude and direction of these responses they induce may be stronger when the birds are exposed to bites from a competent vector.

## 2. Material and methods

An experimental infection was established in 30 uninfected canaries by intraperitoneal injection of approximately 100  µl of *P. relictum* (lineage pSGS1)-infected blood. This lineage was isolated in 2022 from naturally infected canaries (*Serinus canaria*) at Montpellier Zoological Park, cryopreserved immediately, and subsequently used after limited laboratory passage. The inoculum, which was the same for all experimental birds, was prepared by pooling blood from ten donor birds sampled at day 11 post-infection (electronic supplementary material, table S1). Bird parasitaemia was regularly monitored by examining blood smears and counting infected erythrocytes. Experimental birds were randomly assigned to three groups: exposed to the vector species, *Cx quinquefasciatus* (CQ-exposed, *n* = 10), exposed to the non-vector species *An. gambiae* (AG-exposed, *n* = 10) and unexposed (*n* = 10).

Mosquito stimulations were carried out over 3 consecutive days, on days 31–33 post-infection, a period chosen to coincide with the beginning of the chronic stage of the parasite. The mosquito experiments used *Cx. pipiens quinquefasciatus* (SLAB strain) and *An. gambiae* (Kisumu strain). Females of both species were reared in an identical way. Mosquitoes were 7–9 days old, had no prior access to blood, were maintained on 10% glucose solution since emergence, and starved (with access to water) for 6 h before the experiment. All experiments were conducted under controlled insectary conditions (25 ± 2°C, 70% relative humidity, 12:12 light–dark cycle). During the stimulations, birds were individually placed in cages (40 × 30 × 30 cm) with either 30 female *Cx. quinquefasciatus*, 30 female *An. gambiae*, or no mosquitoes for 2 h. To minimize host defensive behaviours, birds were immobilized in a specially designed PVC tube. The number of engorged mosquitoes was recorded after each stimulation period (electronic supplementary material, table S3).

Blood was collected from the brachial vein of experimental birds at six time points: before the first stimulation (D0), 24  h after each mosquito exposure (D1–D3), and 2 and 4 days after the final exposure (D4, D7). For DNA quantification, 10  μl blood was mixed with 100  μl PBS and stored at −20°C; for RNA, 10  μl was mixed with 500  μl Trizol and stored at −80°C. RT-qPCR with newly developed markers quantified male and female gametocytes from RNA, while DNA was used to assess total parasitaemia via established qPCR [35].

To assess whether bites from *Cx. quinquefasciatus* and *An. gambiae* elicit distinct host physiological responses, three independent experiments were conducted, each including 15 birds (five CQ-exposed, five AG-exposed, five controls) and 30 ± 5 mosquitoes per cage. Birds were exposed to mosquitoes over 3 consecutive days under the same experimental conditions as described above. Blood samples were collected before and post-exposure, centrifuged at 3000* g* for 15  min, and plasma was stored at –20°C until analysis. In the first experiment, CRP was measured from 50 µl blood collected daily from D0 to D4 and D7, as acute-phase proteins typically rise within 24 h. IgY was measured from 80 µl blood at D0, D3, D4 and D7, as antibody production occurs on a longer timescale. CRP was measured using a two-site sandwich ELISA (Abebio kit no. AE64569AV) according to the manufacturer’s instructions. Plasma samples for IgY quantification were diluted 1 : 10 (20 μl plasma + 180  μl diluent) prior to analysis. IgY concentrations were measured by Innovative Diagnostics (Montpellier) using a custom anti-IgY ELISA. Plates were coated with 4  µg ml^−1^ anti-chicken antibody in PBS (50 µl well^−1^) overnight at room temperature. Samples were diluted 1 : 5 (50 µl well^−1^), and absorbance was read at 450  nm. Concentrations were interpolated from a standard curve. Corticosterone was quantified in a second experiment. To minimize stress, birds were immobilized in PVC tubes and placed individually in cages at 17.00. They were kept in a quiet darkened room, isolated from external disturbances for 1 h. Samples were taken at D0–D3 and corticosterone levels were measured at the CEBC (Chizé) using an ELISA kit (Demeditec Diagnostics). For this purpose, 50  μl of undiluted plasma per sample was analysed following the manufacturer’s protocol. The assay’s detection limit was 0.204  ng ml^−1^, with an intra-assay coefficient of variation of 5.15%.

### (a) Statistical analyses

To account for baseline variation among birds, parasitaemia and gametocytaemia from D1 onwards were expressed as net changes relative to D0. Conversion rate, the proportion of asexual parasites differentiating into gametocytes, was approximated using the slope of gametocyte abundance over total parasites, since the exact timing of gametocyte development in *P. relictum* is unknown due to overlapping generations. Standard sex ratio (males/total gametocytes) becomes unreliable at low gametocyte densities because minor differences in counts can disproportionately affect estimates. To overcome this, we used the regression slope of male or female gametocytes over total gametocytaemia, providing a more stable measure of sex allocation across conditions, especially in chronic infections. Statistical analyses and graphical representations were performed using the R statistical software (v. 4.3.0). All model specifications are detailed in electronic supplementary material, table S2.

## 3. Results

Infection dynamics before mosquito exposure are shown in electronic supplementary material, figure S1. Parasite dynamics did not differ significantly between treatment groups (model 1, exposure: *χ*²₂ = 0.68, *p* = 0.7121; electronic supplementary material, table S2). Over 90% of mosquitoes fed at each exposure, with no significant difference between the two species (paired *t*‐test: *t* = −1.06, 2 d.f., *p* = 0.40; electronic supplementary material, table S3).

**Figure 1.**
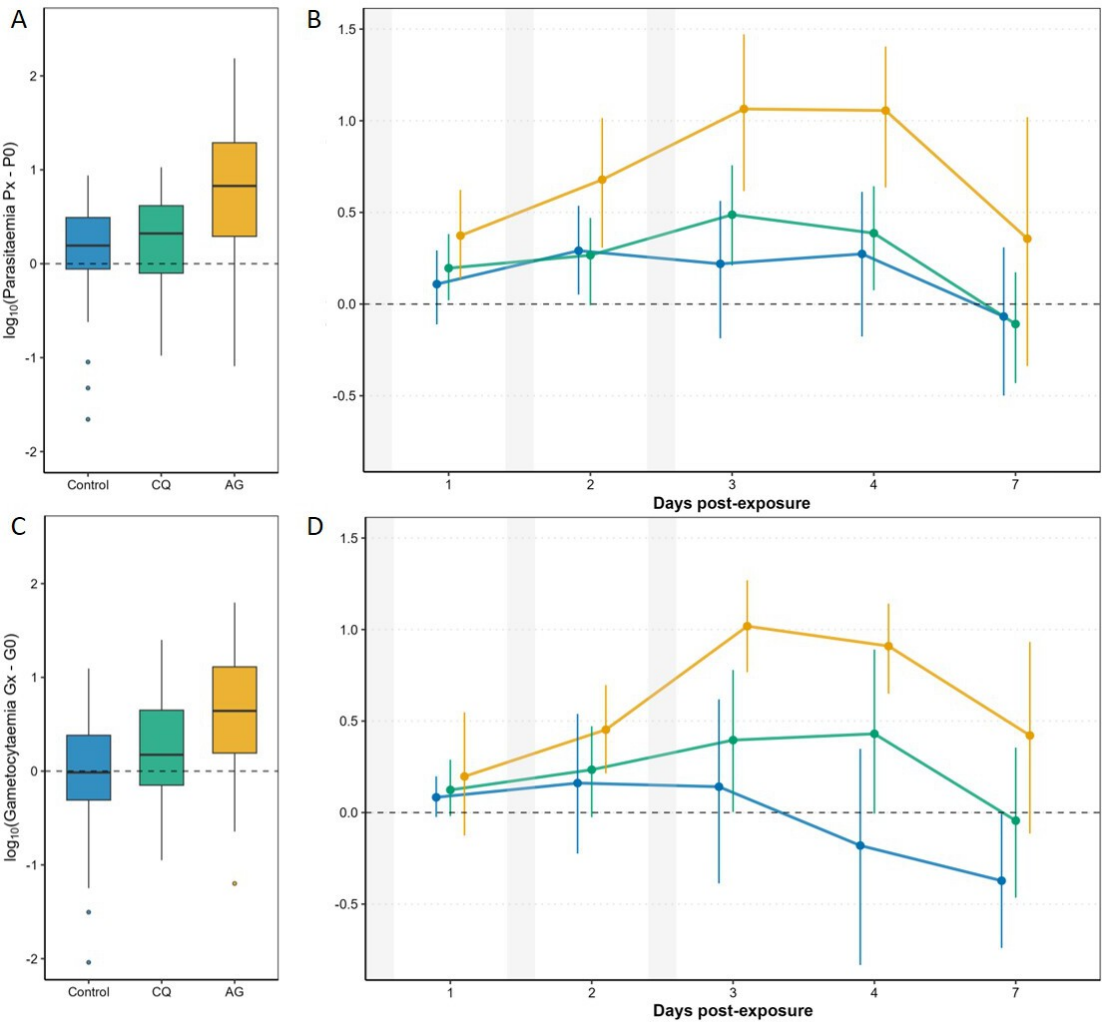
Modulation of *Plasmodium relictum* parasitaemia (A,B) and gametocytaemia (C,D) in response to mosquito exposure. A and C show log-transformed changes across treatments (boxplots: median, IQR, 1.5 × IQR whiskers, outliers). B and D show temporal dynamics (mean ± 95% bootstrapped CI); dashed line marks baseline (31 dpi). Non-overlap with zero indicates *p* < 0.05. Grey shading: mosquito exposure evenings. Blue: unexposed; green: CQ-exposed; yellow: AG-exposed.

The effect of mosquito exposure on parasitaemia and gametocytaemia was assessed using two complementary approaches. First, by comparing within each treatment group (unexposed, CQ-exposed, AG-exposed), the net parasitaemia and gametocytaemia change relative to D0. Second, by performing a full factorial analysis using a mixed-effects model to assess differences in net change in parasitaemia and gametocytaemia across treatments and time points, explicitly testing for interactions between exposure type and day. While parasitaemia in unexposed birds remained unchanged from the pre-exposure baseline, birds exposed to mosquito bites showed a significant increase in parasitaemia, starting 24 h after the initial exposure (D1), which persisted for 3 consecutive days (figure 1B, electronic supplementary material, table S4). Contrary to expectations, this effect was particularly marked for birds exposed to the non-vector species *An. gambiae*. The statistical comparison of the net change in parasitaemias between the treatments revealed a small yet statistically significant effect of mosquito exposure on overall parasitaemia (model 2, *exposure*: χ^2^_2_ = 6.19, *p* = 0.045) which was independent of the sampling time (time*exposure: *χ*^2^_8_ = 11.42, *p* = 0.1788). This effect was largely driven by the marked difference between unexposed and AG-exposed birds: the estimated marginal means (± s.e.), averaged across sampling dates, were nearly fourfold higher in AG-exposed birds (0.71 ± 0.16) compared to unexposed birds (0.18 ± 0.16). In contrast, although parasitaemia in CQ-exposed birds (0.25 ±  0.16) was also higher than in unexposed birds, the difference was not statistically significant (contrast analyses, *p* = 0.965) (figure 1A).

The gametocytaemia results mirrored those of parasitaemia, with a few minor exceptions. Here, the increase in gametocytaemia in birds exposed to mosquito bites occurred at D2 post-exposure, i.e. 24 h after the first differences in parasitaemia were observed. The largest increases in gametocytaemia were also observed in AG-exposed birds (figure 1D). Gametocytaemia in unexposed birds remained at the pre-exposure baseline until the final sampling day (D7), at which point it decreased significantly. The statistical comparison of net changes in gametocytaemia between treatments revealed a highly significant interaction between exposure and sampling day (model 3, time*exposure: *χ*^2^_8_ = 23.946, *p* = 0.0023) (figure 1C). Contrast analyses revealed significant differences between unexposed and AG-exposed birds at D3 (*p* = 0.007), D4 (*p* = 0.0008) and D7 (*p* = 0.023), whereas no significant differences were observed between unexposed and CQ-exposed birds at any time point (figure 1D).

**Figure 2.**
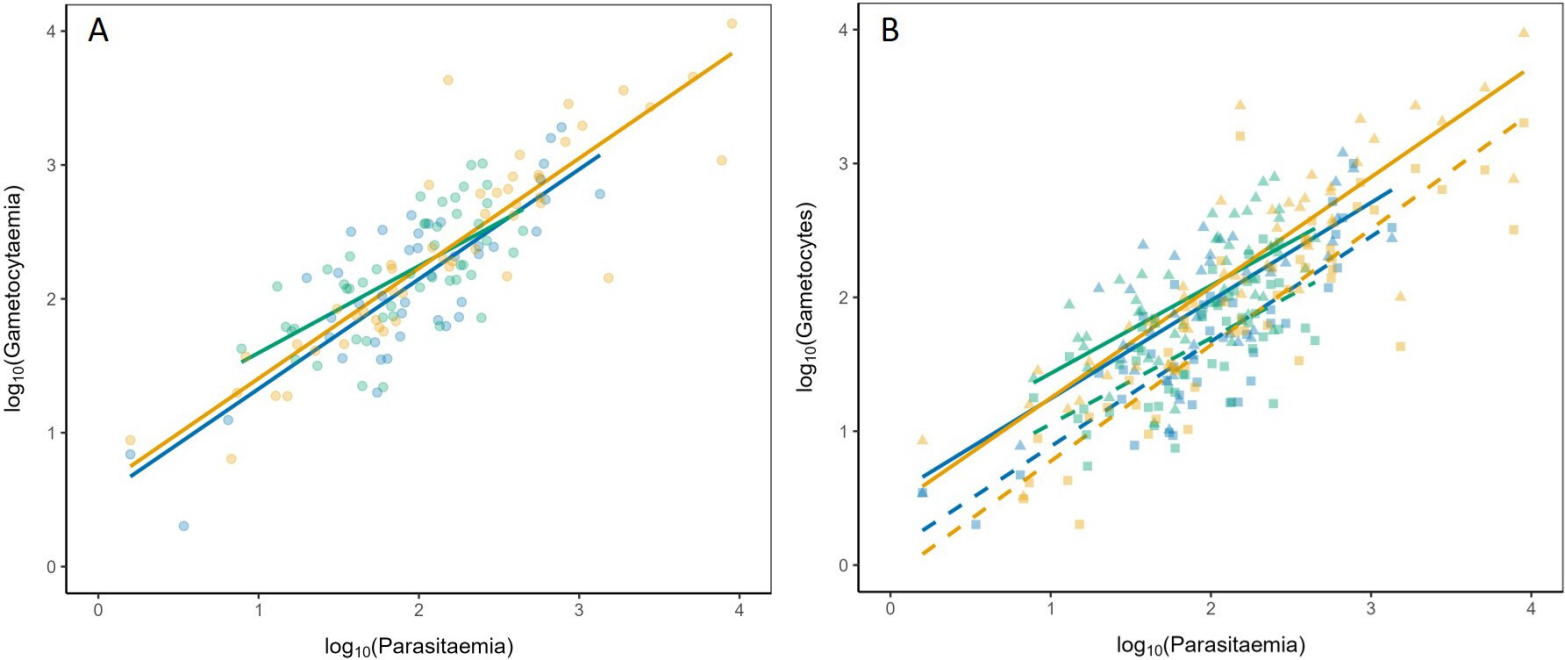
Gametocyte conversion rate (A) and sex allocation dynamics (B) in unexposed (blue), CQ-exposed (green) and AG-exposed (yellow) birds. In (B), females = solid line and triangles, males = dashed line and squares.

Gametocytaemia was strongly and positively correlated with parasitaemia (*parasitaemia: χ*^2^_1_ = 111.87, *p* < 0.001). There were, however, no significant differences between the slopes of the regression among the three different exposure treatments (model 4, parasitaemia*exposure: *χ*^2^_2_ = 2.35, *p* = 0.3082) (figure 2A). In other words, the conversion rate remained consistent across the three different exposure treatments [2,3]

Female gametocytes were generally more abundant than male gametocytes across treatments (*sex*: *χ*^2^_1_ = 106.4, *p* < 0.0001) (electronic supplementary material, figure S2). However, mosquito bites did not significantly affect investment in male or female gametocyte production, as there was no significant difference between the slopes for male and female gametocytes (sex*parasitaemia: *χ*^2^_2_ = 1.0911, *p* = 0.2962), nor between exposure groups (model 5, sex*exposure: *χ*^2^_1_ = 2.8117, *p* = 0.2452) (figure 2B).

**Figure 3.**
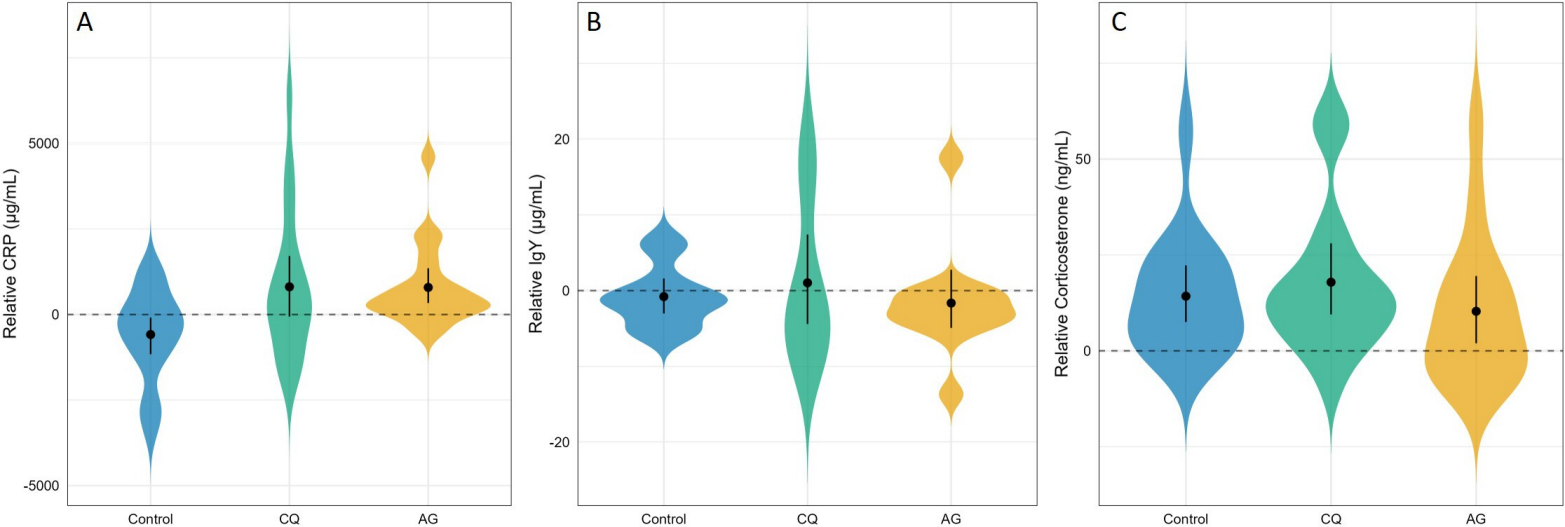
Relative levels of physiological and immune biomarkers post-exposure: (A) CRP, (B) IgY, (C) corticosterone in unexposed (blue), CQ-exposed (green), and AG-exposed (yellow) birds. Violin plots show value distributions; black bars = bootstrapped mean ± 95% CI. Dashed lines = D0 baseline.

Exposure to mosquito bites induced an increase in circulating CRP levels, which was independent of the sampling time (model 6, *exposure*: *χ*² = 6.52, *p* = 0.038; *time*: *χ*² = 8.70, *p* = 0.0688, figure 3A). However, *post-hoc* comparisons revealed no significant difference between the two mosquito species (*p* = 0.99). IgY levels were unaffected by mosquito exposure (model 7, *exposure: χ*² = 0.31, *p* = 0.86; figure 3B) and remained stable over time (model 7, *time: χ*² = 0.59, *p* = 0.7400). Similarly, relative corticosterone levels did not differ between treatment groups (model 8, *exposure*: *χ*² = 1.28, *p* = 0.52; figure 3C), nor did they exhibit any significant temporal change (*time*: *χ*² = 0.21, *p* = 0.901).

## 4. Discussion

Previous studies have reported significant increases in blood parasitaemia and infectivity to mosquitoes in *P. relictum*-infected birds exposed to *Cx. pipiens* bites, albeit in birds with late chronic infections (older than 60 days post-infection [11,14]). Here, we exposed canaries with early chronic infections (approx. 31 days post-infection) to bites from *Cx. quinquefasciatus*, a natural vector of *P. relictum*, and from *An. gambiae*, a non-vector species with no known history of sympatry or transmission compatibility with this parasite lineage. Our results show an increase in parasitaemia following exposure to bites from both mosquito species (figure 1, electronic supplementary material, table S2). In particular, birds exposed to *Cx. quinquefasciatus* and *An. gambiae* exhibited significantly higher parasitaemia on D1–D4 compared to their pre-exposure baseline, a pattern that was not observed in unexposed controls. However, the full factorial mixed-effects model comparing treatments across time points did not identify a statistically significant difference between the CQ-exposed and unexposed groups. This apparent discrepancy likely arises from the differing sensitivities of within-group and between-group analyses: while within-group comparisons detect temporal shifts relative to baseline, the mixed model accounts for temporal variability across all groups, offering a more conservative test of treatment effects. A similar pattern was observed for gametocytaemia, which increased more markedly in AG-exposed birds, whereas in CQ-exposed birds, only a marginal and transient increase was detected on days 3 and 4 post exposure.

The stronger response of *P. relictum* to *An. gambiae* was unexpected given that this parasite is not a competent vector. Nevertheless, such a generalist response could be adaptive if non-competent mosquitoes serve as probabilistic proxies of vector presence or provide anticipatory cues of transmission opportunities. Mosquito feeding rates were equally high for both species, ruling out differences in exposure frequency. However, blood meal size was not quantified, leaving potential differences unverified. We hypothesized that the novelty of *An. gambiae* for canaries, two species that do not overlap geographically, may have elicited a heightened physiological reaction in the bird, to which the parasite responded. Mosquito saliva contains a complex mixture of pharmacologically active components, including anti-coagulants, vasodilators, immunomodulators and anti-inflammatory molecules, which differ between species [36,37] and may thus trigger different host immune and neuroendocrine responses [38]. Notably, though, CQ- and AQ-exposed birds exhibited comparable levels of inflammatory (CRP), humoral (IgY) and physiological stress (corticosterone) markers. These markers were chosen to provide a broad assessment of the host’s systemic response to mosquito bites. CRP is a widely used indicator of acute inflammation [32,39], while IgY levels serve as a proxy for the bird’s adaptive humoral immune response, offering insight into longer-term or antigen-specific immunological activation [33]. In mammals, mosquito bites can enhance IgE titres [40,41], suggesting that the functionally analogous IgY [42] may play a comparable role in avian anti-vector responses. Corticosterone, the primary avian glucocorticoid, was measured to assess the physiological stress response, and previous work has shown that *P. relictum* reacts to elevated corticosterone levels by increasing its parasitaemia and gametocytaemia [34]. However, it remains possible that other, unmeasured, physiological pathways were involved. Future work should broaden the scope of host response characterization by incorporating additional immune and endocrine markers as well as parasite transcriptomic profiling to better elucidate the mechanisms underlying species-specific responses to mosquito bites.

By using recently developed molecular tools that allow more precise quantification of male and female gametocytes [27], we were able to overcome the limitations of conventional microscopy to quantify not only total parasitaemia but also the estimated number and sex of gametocytes. Previous studies in human (*P. falciparum*) and rodent (*P. chabaudi*) malaria parasites have demonstrated plasticity in gametocyte production and sex ratio in response to changes in host immunity [43–45], anaemia [46–49] and drug treatment [50–53]. In contrast, our results indicate that *P. relictum* does not modulate either gametocyte conversion rate or sex ratio in response to mosquito exposure, suggesting a lack of short-term plasticity in these key transmission traits. These findings suggest that, in this system, transmission-stage investment is tightly coupled to asexual replication dynamics, and that environmental responsiveness operates through adjustments in overall parasite burden rather than targeted shifts in gametocytogenesis.

However, a crucial difference between *P. falciparum* and *P. chabaudi*, on the one hand, and *P. relictum*, on the other hand, is that the former are synchronous parasites [54], while *P. relictum* exhibits asynchronous development. This distinction has important implications for detecting plastic responses in transmission investment. In synchronous parasites, non-overlapping cohorts allow clear temporal resolution of developmental events, making it easier to link changes in gametocyte production to specific environmental cues such as mosquito bites. In contrast, the continuous production and maturation of blood stages in asynchronous parasites like *P. relictum* create overlapping generations that can dilute or obscure short-term shifts in gametocyte commitment. Additional factors, such as the dynamics of asexual replication, erythrocyte availability and gametocyte lifespan, may further blur estimates of gametocyte conversion rates [55]. As a result, the apparent lack of plasticity in gametocytogenesis observed in our study may reflect not a true absence of response, but the limitations of detecting such changes in a complex system.

In conclusion, these findings, together with previous work [11,12,56], argue in favour of a generalist response strategy in *P. relictum*, whereby the parasite is capable of reacting to both vector and non-vector species by increasing its parasitaemia and gametocytemia. While seemingly inefficient, this strategy may be adaptive in ecological contexts where different mosquito species exhibit overlapping temporal and spatial distributions. For generalist parasites with broad host or vector ranges, such as *P. relictum* [25,57], treating any mosquito bite as a probabilistic cue of transmission opportunity may represent an effective bet-hedging strategy. These results warrant further investigation across a broader range of vector and non-vector mosquito species, as well as different *Plasmodium* lineages and host systems, to assess the generality of the observed patterns. Should similar responses be observed in other contexts, it would raise important considerations for avian malaria management and disease forecasting. If non-vector mosquitoes can amplify transmission investment in *Plasmodium*, shifts in mosquito community composition, driven by climate change, urbanization or vector control campaigns [58–60] could unintentionally affect parasite dynamics.

## Data Availability

The datasets supporting this article have been deposited in the Dryad Digital Repository [61]. Supplementary material is available online [62].
